# The Corrosion Probability and Flexural Strength of an RC Beam under Chloride Ingress Considering the Randomness of Temperature and Humidity

**DOI:** 10.3390/ma13102260

**Published:** 2020-05-14

**Authors:** Sen Pang, Ming-kai Yu, Hong-guang Zhu, Cheng Yi

**Affiliations:** School of Mechanics and Civil Engineering, China University of Mining and Technology-Beijing, Beijing 100083, China; pang.sn@buaa.edu.cn (S.P.); sqt1700602053@student.cumtb.edu.cn (M.-k.Y.); yc@cumtb.edu.cn (C.Y.)

**Keywords:** chloride, randomness of temperature and humidity, Monte Carlo method, flexural strength, probability model, offshore concrete

## Abstract

The load capacity of reinforced concrete structure will decrease by chloride ingress in coastal region. In this paper, the corrosion probability and flexural strength of a typical reinforced concrete beam design under the influence of temperature and humidity was obtained by the Monte Carlo method. The relationship between flexural strength, temperature, relative humidity, and geometric parameters was established. The annual average temperature and relative humidity were treated as random variables together with the geometric size and concrete compressive strength. The corrosion probability and flexural strength in a wave splashing zone, coastal atmospheric zone, and offshore atmospheric zone were calculated. The results show that the corrosion probabilities of the three regions are obviously different. When the standard deviation of temperature is less than 1.5 °C, the temperature can be treated as a constant in the calculation of the concrete cracking probability in the wave splashing zone. A binary logistic regression formula was given to predict whether the randomness of temperature and humidity should be considered in the offshore atmospheric zone. When the standard deviation of the temperature is less than 1 °C, the temperature randomness has no significant effect on the flexural strength of beams in the wave splashing zone. The flexural strength distribution conforms to the normal distribution in the early stage of service and the Weibull distribution after concrete cracking.

## 1. Introduction

Coastal reinforced concrete structures will be affected by chloride ingress, resulting in the decline of load bearing capacity. The process of chloride ingress and steel corrosion involves complex chemical reactions and mass transfer, which is affected by ambient temperature and humidity. Due to the randomness of the variables, it is difficult to accurately calculate the degradation of the structure load bearing capacity. Instead, the probability of the decline of the structure load bearing capacity is more concerned. However, numerous challenges remain in obtaining the probability. For instance, although the probability distributions of basic variables, such as temperature and humidity, have been investigated by many studies, it is very difficult to obtain the probability distribution of load bearing capacity by analytical method because of the complexity of the formulas in calculation. Moreover, it is infeasible to obtain the probability distribution of the load bearing capacity directly by experimental methods, which will cost a large amount of resources. Many studies [1,2,3,4,5] have been conducted on chloride ion diffusion and steel corrosion using experimental or analytical methods, which provide the basis of probability calculation. Bohni [6] comprehensively explained the basic corrosion mechanisms in reinforced concrete structures. When the probability distribution of basic parameters is known, the distribution of the load bearing capacity can be calculated by Monte Carlo method. Some studies investigated the statistical distribution of basic parameters. Masi et al. [7,8] analyzed the inter- and intra-variability of the concrete strength of existing buildings using a very large database. Results show that the CV of concrete strength varies from different storeys. Kilinc et al. [9] and Celik et al. [10] investigated the statistical distributions of in situ microcore concrete strength and the distribution is a log-normal distribution. In other studies [11,12,13,14,15,16,17], the probabilistic approaches were performed to predict the service life of structures by establishing the relationship between different factors and chloride diffusion. Most studies [18,19,20,21] calculated chloride diffusion based on Fick’s second law but the random temperature and humidity make the partial differential equation very difficult to solve. Few studies focus on random changes of temperature and humidity.

The Monte Carlo method has advantages in high-dimensional problems and complex computation, which can calculate complex problems through simple programming and the computation speed is rather fast due to modern computer technology. In this study, the Monte Carlo method was applied to calculate the probability distribution of flexural strength of reinforced concrete beam. In order to obtain the probability model of load bearing capacity, the relationship between basic variables, such as temperature and humidity, and load bearing capacity needs to be established. Moreover, the random distribution of basic variables is required to apply the Monte Carlo method. Collepardi et al. [22] first used Fick’s second law to describe the diffusion of chloride ions in concrete. Since the reinforced concrete structure is in a complex and changeable environment, the diffusion behavior of chloride ions in concrete is affected by various factors. On this basis, Fick’s second law was modified and a multi-coefficient model was established by many researchers. Tang and Nilsson [23] believed that the chloride diffusion coefficient had a certain relationship with time and established a time-varying diffusion model. Thomas and Bamforth [24] improved the time-varying diffusion model by considering the effect of fly ash and slag. Liu et al. [25] proposed a new quantified model to investigate chloride diffusion in the offshore concrete structures. Saetta et al. [26] studied the effect of humidity on chloride diffusion and determined the humidity effect coefficient. The effect of temperature on chloride diffusion has been studied by many studies [27,28], and results show that the effect of temperature can be described by the Arrhenius formula. Liu et al. [29,30,31,32] studied the transport mechanism of chloride ion in concrete comprehensively and established a multi-component ionic transport model. Jiang et al. [33,34] studied the diffusion of chloride in concrete affected by freeze-thaw. Shen et al. [35,36] studied the effect of concrete carbonation on chloride binding and diffusion in concrete.

Chloride ingress will eventually lead to steel corrosion, which is the main reason for the decline in load bearing capacity. The depth of corrosion (radius loss) and the density of corrosion current are generally used to evaluate the degree of steel corrosion. Bazant and Zdenek [37] established a set of differential equations based on the mass conservation of each reactive substance in the process of steel reinforcement corrosion, the first diffusion law of Fick, the Maxell electrostatic equation, and the chemical reaction rate equation, and provided the physical model of steel reinforcement corrosion according to the characteristics of concrete structures in the marine environment. Research [15,38] established the relationship between corrosion current density and time by experimental data. Zhang et al. [39] analyzed the cracking process of concrete caused by uneven corrosion. Val et al. [40] proposed a hemispherical pit corrosion model for local corrosion of reinforcement caused by chloride ions.

In this paper, based on the studies of influence of temperature and humidity on chloride penetration, the relationship between environment parameters and steel corrosion was deduced, and then the model of load bearing capacity under the influence of temperature and humidity was obtained. The experimental data in different literatures were compared to verify the accuracy of the model. The random distributions of parameters in different literatures were investigated. Through programming to generate random parameters, the corrosion probability and the law of flexural strength decline changes with time were obtained by the Monte Carlo method. The results of temperature and humidity as random variables and constants are compared. The effects of variance of different parameters on the corrosion probability and distribution of flexural strength were discussed. A binary logistic regression formula was proposed to predict whether temperature and humidity randomness should be considered.

## 2. Methods

### 2.1. Steel Bar Corrosion Model under Chloride Ingress

#### 2.1.1. Diffusion Coefficient of Chloride Ions Affected by Temperature and Humidity

The corrosion of steel bar in concrete under chloride ingress is usually divided into three stages: the corrosion initiation stage, corrosion propagation stage and expansion failure stage. The corrosion initiation stage is the process in which chlorine ions diffuse into the concrete and accumulate to the threshold value of reinforcement depassivation. During this period, it is believed that the reinforcement is not corroded. The duration of corrosion initiation stage, *t_i_*, is usually calculated by Fick’s second law as:

(1) $$\frac{\partial C}{\partial t}=D(t)\frac{\partial C^{2}}{\partial^{2}x}$$

where *C* is the chloride concentration, *D(t)* is the chloride diffusion coefficient, *x* is depth in concrete and *t* is time.

In Tang and Nilsson’s [23] and Life-365 program [41], the results show that chloride diffusion coefficient has a certain relationship with time and expressed the phenomenon that the diffusion coefficient of chlorine ions decreased with the increase of time by power function, as shown in Equation (2).

(2) $${D(t)=D}_{0}{\left(\frac{t_{0}}{t}\right)}^{m}$$

where *D_0_* is the chloride diffusion coefficient at time *t_0_*, *t_0_* is the reference time from concrete pouring (usually is 28 d), *D*_0_ is expressed as $D_{0}{=10}^{(-12.06+2.4w/c)}$. in Life-365 program which is at saturation. *m* is the diffusion decay factor, m=0.2+0.4(FA/50+SG/70). [24], where *FA* and *SG* are, respectively, the proportion of fly ash and slag in the mixture. The chloride diffusion coefficient is affected by temperature and relative humidity. In [26,42,43], the multifactor chloride diffusion coefficient is calculated by:

(3) $${D(t)=k}_{T,cl}k_{H,cl}D_{0}{\left(\frac{t_{0}}{t}\right)}^{m}$$

where *k_T,cl_* is the temperature effect coefficient in chloride ingress, *k_H,cl_* is the humidity effect coefficient in chloride ingress. For the calculation of *k_T,cl_*, the effect of temperature on chloride diffusion coefficient can be described by Arrhenius’ law based on the results of [27,28]:

(4) $$k_{T,cl}{=e}^{b_{e}(\frac{1}{T_{0}+273}\;-\;\frac{1}{T+273})}$$

where *b_e_* is the regression coefficient and is normally distributed. Based on a large number of experimental data statistics, the mean of *b_e_* is 4758.47 and the variance is 798.132. *T_0_* is the reference temperature and is usually 20 °C. *T* is the environment temperature (°C).

For the calculation of *k_H,cl_*, the humidity effect coefficient is expressed by studies [26,44] as:

(5) $$k_{H,cl}={\left[1+\frac{{{(1-RH}_{c})}^{4}}{{{(1-RH}_{0})}^{4}}\right]}^{-1}$$

where *RH*_0_ is the critical relative humidity which is 0.75. *RH*_c_ is the relative humidity of environment.

**Figure 1:**
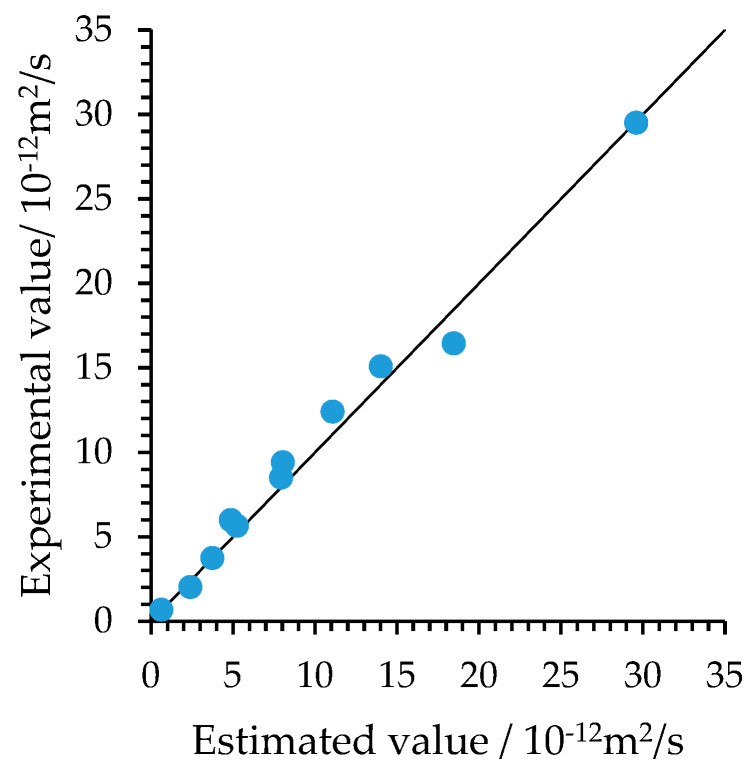
Comparison between experimental values and estimated values.

In order to validate the accuracy of Equation (3), the experimental values in studies [45,46,47,48] and estimated values obtained by (3) are compared. The comparison results are show in Figure 1 and Table 1.

#### 2.1.2. Corrosion Initiation Time and Concrete Cover Cracking Time

Equation (1) is a nonlinear partial differential equation. To solve Equation (1), the variable substitution is used as follows where *t*_0_ is the age of concrete when chloride ion ingress begins:

(6) $$\;S(t)=\int_{t_{0}}^{t}k_{T,cl}k_{H,cl}D_{0}{\left(\frac{t_{0}}{t}\right)}^{m}dt$$

(7) $$\frac{dS\left(t\right)}{dt}{=k}_{T,cl}k_{H,cl}D_{0}{\left(\frac{t_{0}}{t}\right)}^{m}$$

According to the derivation rule of compound function:

(8) $$\frac{\partial C}{\partial t}=\frac{\partial C}{\partial S}\cdot \frac{dS}{dt}{=k}_{T,cl}k_{H,cl}D_{0}{\left(\frac{t_{0}}{t}\right)}^{m}\cdot \frac{\partial C}{\partial S}$$

Equation (9) can be obtained by comparing Equations (1) and (8):

(9) $$\frac{\partial^{2}C}{\partial x^{2}}=\frac{\partial C}{\partial S}$$

Considering three boundary conditions, 1.The concentration of chloride ion on concrete surface is constant, *C*(*x* = 0,*t*) = *C_S_*; 2.The concentration of chloride ion in concrete is *C*_0_ at the initial time, *C*(*x*,*t* = 0) = *C*_0_; 3.The concentration of chloride ion in concrete is *C*_0_ at infinity, *C*(*x* = ∞,*t*) = *C*_0_, a special solution can be obtained from Equation (9):

(10) $${C(x,t)=C}_{0}{+(C}_{s}-C_{0})\left[1-erf\left(\frac{x}{2\sqrt{S\left(t\right)}}\right)\right]$$

When chloride concentration reaches the chloride threshold *C_t_*, the steel bar in concrete begins to rust. Substituting concrete cover thickness for *x*, and *C_t_* for *C*(*x*,*t*) in Equation (10), the corrosion initiation time *t_i_* can be obtained.

When the steel bar begins to rust for a period of time, the concrete cover will crack due to the accumulation of corrosion products. This period is the concrete cover cracking time, *t_c_*. In the standard for durability assessment of existing concrete structures (GB/T 51355-2019) [49], the formula for calculating the critical corrosion depth of reinforcement is expressed as:

(11) $$\delta_{cr}=0.012\frac{c}{d}+0{.00084f}_{cu,k}+0.018$$

where *d* is the diameter of steel bar(mm), *c* is concrete cover thickness (mm), and *f_cu,k_* is the standard value of the compressive strength of concrete (MPa).

Based on Faraday’s law, the corrosion rate of metal is obtained from corrosion current density:

(12) $$\lambda =\frac{3{.27\;\times \;10}^{-3}i_{corr}M}{n\rho }$$

where *λ* is the metal corrosion rate(mm/a), *i_corr_* is the corrosion current density(*μ*A/cm^2^), *M* is the relative atomic mass, *n* is the metal valence and *ρ* is the metal density. The following expression of metal corrosion rate before concrete cover cracking can be obtained by substituting the parameters of iron. Steel bars of different strength grades have the same density and metal valence. The corrosion rate can be calculated by the same expression:

(13) $$\lambda_{1}=0{.01166i}_{corr}$$

*λ*_1_ is the metal corrosion rate before concrete cover cracking. Liu et al. [38] obtained the corrosion rate of a steel bar before concrete cover cracking by fitting with relevant data. Vu et al. [15] proposed a new equation of corrosion current density based on Liu et al.’s research. In the standard for durability assessment of existing concrete structures (GB/T 51355-2019) [49], an equation was proposed considering temperature and humidity based on Liu et al.’s research and survey data in an actual structure:

(14) $${ln\;i}_{corr}=8.617+0{.618lnC}_{sl}-\frac{3034}{T+273}{-5\;\times \;10}^{-3}{\rho +ln\;m}_{cl}\;{\rho =k}_{\rho }(1{.8-C}_{cl}^{\mu }{)+10(RH-1)}^{2}+4$$

where *C_sl_* is the chloride concentration on steel bar surface (kg/m^3^). *T* is the temperature (°C). *ρ* is the concrete resistivity (kΩ·cm). *m_cl_* is the local environmental coefficient and the value is 4.5~5.5 in the wave splashing zone and outdoor wet-dry cycling environment, and 4.0~4.5 in hot and humid regions. *K_ρ_* is coefficient. When water to cement ratio is 0.3~0.4, *K_ρ_* is 11.1. When the water-to-cement ratio is 0.5~0.6, *K_ρ_* is 5.6. $C_{cl}^{\mu }$ is the average concentration of chloride in concrete cover (kg/m^3^). When $C_{cl}^{\mu }$ > 3.6, $C_{cl}^{\mu }$ = 3.6. RH is the relative humidity. The average depth of steel corrosion at *t* after corrosion initiation is:

(15) $$\delta_{av}\left(t\right)=\int_{0}^{t}\lambda_{1}dt=0{.01166i}_{corr}\cdot t$$

When the average depth of steel corrosion reaches the critical corrosion depth *δ_cr_*, the concrete cover will crack. The concrete cover cracking time, *t_c_*, can be calculated by substitute (11) into (15):

(16) $$t_{c}=\frac{0.012\frac{c}{d}+0{.00084f}_{cu,k}+0.018}{0{.01166i}_{corr}}$$

#### 2.1.3. Maximum Section Loss Rate of Steel Bar

The loss of steel bar section area can be calculated using the corrosion depth. The section loss rate of a steel bar is defined as:

(17) $$\eta (t)=\frac{A_{corr}\left(t\right)}{A_{0}}$$

where *A*_0_ is the initial sectional area of steel bar and *A_corr_* is the corrosion area of steel bar. In the present study, the calculation formulas for the section of corroded steel bar mainly include uniform corrosion model and pitting corrosion model. Chloride ions usually reach the steel bar surface quickly through concrete cracks and then adsorb on the local passivation film, which causes the reinforcement serious local corrosion to form the pitting corrosion. Therefore, the pitting corrosion model was introduced in this study to research the section loss of steel bar. In order to obtain the maximum section loss rate, the non-uniform coefficient *R* is introduced to describe the maximum depth of pitting corrosion:

(18) $$\delta_{max}\left(t\right){=R\delta }_{av}\left(t\right)$$

By substituting Equation (15) into Equation (18), the expression of *δ_max_(t)* can be obtained:

(19) $$\delta_{max}\left(t\right)=\{\begin{array}{ll} 0 & {t\;<\;t}_{i}\\ {R\lambda }_{1}{(t-t}_{i}) & t_{i}\;\leq {\;t\;<\;t}_{i}{+t}_{c}\\ {R\lambda }_{1}\left({t-t}_{i}\right){+R\lambda }_{2}{t-t}_{i}{-t}_{c} & t\;\geq {\;t}_{i}{+t}_{c} \end{array}$$

**Figure 2:**
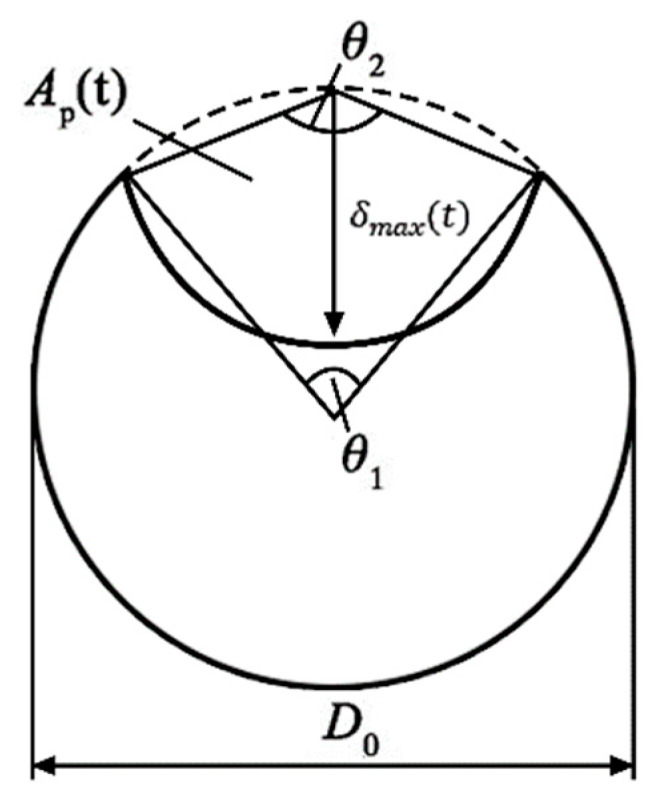
Diagram of pitting corrosion area of a steel bar.

The value of *R* is 4 in this study based on the [50]. The λ_2_ is the corrosion rate of the steel bar after concrete cover cracking and can be calculated as $\lambda_{2}=\left(4{.5-26\lambda }_{1}\right)\lambda_{1}$. When *λ*_2_ < 1.8*λ*_1_, *λ*_2_ = 1.8*λ*_1_ [51]. The maximum section loss rate of steel bar is determined by hemispherical pitting corrosion model as shown in Figure 2. The pitting corrosion area, *A_p_*(*t*), can be calculated from the size of geometry:

(20) $$A_{p}\left(t\right)=\{\begin{array}{ll} A_{1}{+A}_{2} & \delta_{max}\left(t\right)\;<\;\frac{d}{\sqrt{2}}\\ \frac{{\pi d}^{2}}{4}{-A}_{1}{+A}_{2} & \frac{d}{\sqrt{2}}\;\leq {\;\delta }_{max}\left(t\right)\;\leq \;d\\ \frac{{\pi d}^{2}}{4} & \delta_{max}\left(t\right)\;>\;d \end{array}$$

$$A_{1}=0.5\left[\theta_{1}{\left(0.5d\right)}^{2}-a\left|0.5d-\frac{\delta_{max}^{2}\left(t\right)}{d}\right|\right]{;\;A}_{2}=0.5\left[\theta_{2}\delta_{max}^{2}\left(t\right)-\frac{{a\delta }_{max}^{2}\left(t\right)}{d}\right];\;\;{a=2\delta }_{max}\left(t\right)\sqrt{1-[\frac{\delta_{max}\left(t\right)}{d}{]}^{2};}\theta_{1}=2arcsin(\frac{a}{d}{);\theta }_{2}=2arcsin[\frac{0.5a}{\delta_{max}\left(t\right)}]$$

### 2.2. Flexural Strength of Reinforced Concrete Beam

The ultimate flexural capacity of the reinforced concrete beam under a chloride environment would decrease due to the corrosion of steel bars. By substituting the corroded steel section area for the initial steel section area, the flexural capacity of reinforced concrete beam only with tension reinforcement can be calculated as follows:

(21) $$\{\begin{array}{c} \alpha_{1}f_{c}{\mathrm{bx}=(1-\eta )f}_{y}A_{s}\\ M_{u,corr}{=\alpha }_{1}f_{c}{\mathrm{bx}(h}_{0}-\frac{x}{2}) \end{array}$$

where *f_y_* is the yield strength of steel bar, *η* is the section loss rate of steel bar. *A_s_* is the total section area of steel bars, *b* is the breadth of beam, *x* is the height of equivalent rectangular compression zone, *h_0_* is the effective section height, *α*_1_ is the graphics coefficient, which is applied to calculate the equivalent stress of concrete in equivalent rectangular compression zone, and usually is 0.8 according to the code for the design of concrete structures (GB 50010-2002) [52].

By simultaneously solving Equation (21), the ultimate flexural capacity of the reinforced concrete beam is obtained:

(22) $$M_{u,corr}{=(1-\eta )f}_{y}A_{s}\left[h_{0}-\frac{{(1-\eta )f}_{y}A_{s}}{{2\alpha }_{1}f_{c}b}\right]$$

## 3. Calculation of Monte Carlo Method

### 3.1. Example Parameters

**Figure 3:**
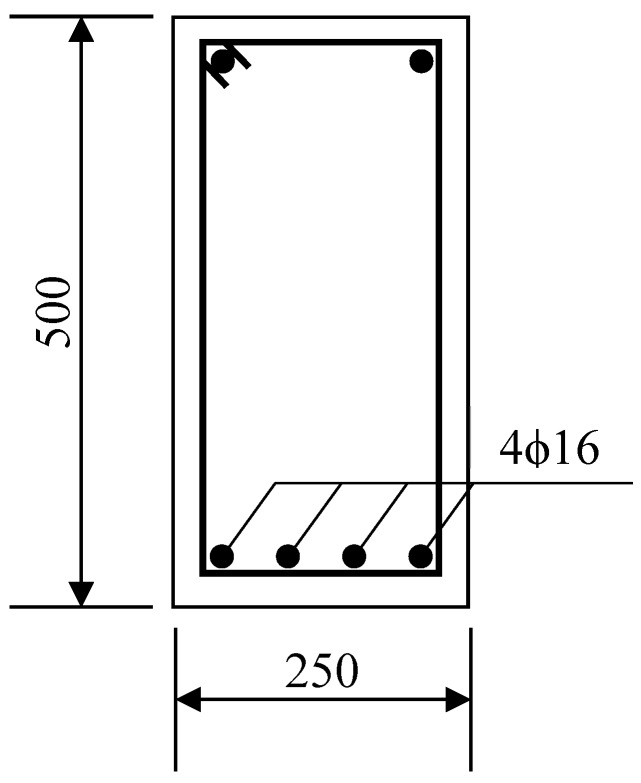
Reinforcement figure of the beam in calculation (unit: mm).

Qingdao is located on the northern coast of China. Taking a rectangular beam with tension reinforcement in Qingdao as an example, the effects of temperature and humidity randomness on flexural strength was calculated by the Monte Carlo method. Material parameters and the beam reinforcement configuration are shown in Table 2 and Figure 3. The top bars in Figure 3 are used to support the stirrup and are neglected in the flexural strength calculation. The effect of concrete load cracking on chloride ion diffusion is neglected here.

The main sources of chloride ions on the surface of reinforced concrete structures near the coast are seawater and salt spray. The chloride content of the concrete surface (*C_s_*) is different according to the different environment. Three different regions are considered in this paper, which are the wave splashing zone, the coastal atmospheric zone, and the offshore atmospheric zone. The coastal atmosphere is defined as an area above sea level where there is no direct contact with seawater but is affected by salt spray. The offshore atmospheric zone is defined as an area 0.5 km from the coast. Different standards and studies have different recommendations for the value of *C_s_*. The values of *C_s_* in different standards are listed in Table 3. The unit of all the values in standards and studies has been converted into kg/m^3^, which is the chloride ion mass per unit volume of concrete (approximately, concrete density is 2300 kg/m^3^ and dosage of cementing material is 400 kg/m^3^, corresponding to concrete grade C40). ACI-365 and JSCE consider the effect of distance from the coast on chloride concentration. ACI-365 suggests that the surface chloride concentration in the coastal zone is accumulated per year and the final value is listed in Table 3. The annual average of the coastal zone and offshore zone are 11.5 and 6.9, respectively. The report [53] of 13 cross-sea bridges in Florida shows the surface chloride concentration of splash zone is 18 kg/m^3^. In the 1990s, Norway [54,55] conducted several large-scale surveys of existing projects, including the undersea tunnel, the North Sea oil platform, and bridges. The results showed that the *C_s_* of bridge in atmospheric zone would accumulate to the same value of splash zone. The coastal region is zoned simply by DuraCrete [56], hence the values in coastal atmospheric zone and offshore atmospheric zone in Table 3 are the same. The surface chloride concentration in the wave splashing zone was investigated in Chinese ports which were in service for 9~16 years and the value of *C_s_* ranges from 1.7~18 kg/m^3^ [49]. Bamforth [57] investigated the chloride content on concrete surfaces of the British coast and presented the design values of *C_s_* without the distance from the coast. McGee [58] conducted a field-based study of 1158 bridges in the Australian state of Tasmania and the surface chloride concentration in the atmospheric zone is expressed as a function of distance from the coast. The mean values of *C_s_* in study [58] are listed in Table 3.

Most investigations indicate that the surface chloride concentration in the splash area which directly contacts with seawater is close to 18 kg/m^3^. The value in atmospheric zone above the sea varies greatly according to the wind direction and height above sea level, but it will accumulate eventually to the same value of splash zone. In the region with a certain distance from the coast, which is usually 1~3 km, the content of chloride accumulated on the surface decreases significantly. Unfortunately, the data for coastal atmospheric zone is very limited. Therefore, the value of *C_s_* in the wave splashing zone is 18 kg/m^3^ in the calculation of this paper according to the most surveys. Although the value in atmospheric zone above sea will accumulate to the same value as the splash zone, a value less than that in splash zone is used in the calculation for the coastal atmospheric zone, which is 11.5 kg/m^3^, considering the time of build-up. This value is the mean value in the accumulation progress suggested by ACI-365 and is relatively close to the recommended value of JSCE and Bamforth. Due to the lack of sufficient survey data, the value in the offshore atmospheric zone applied in the calculation of this paper is 3 kg/m^3^ only for comparison purpose.

The critical threshold concentration of chloride, *C_t_*, varies with different environment and is distributed between 0.3~2.4 kg/m^3^. The value of *C_t_* which is commonly used for service life prediction purposes is the value of 0.40 percent (chloride based on the mass of cementitious materials). In this paper, the value of 1.5 kg/m^3^ is applied in the calculation.

### 3.2. Randomness of Different Parameters

The randomness of the temperature and humidity and other parameters, such as the concrete cover thickness, affect the flexural strength of reinforced concrete beams. The temperature and humidity are considered constant throughout the service life of structures in the standard for durability assessment of concrete structures. However, many studies suggest that temperature and humidity should be treated as random variables. In this paper, the average annual temperature and humidity are treated as random variables, which means that the temperature and humidity are averaged throughout the year, but vary from year-to-year. Therefore, the temperature effect coefficient and the humidity effect coefficient are piecewise functions of time. Different temperature and humidity values will result in different *k_T_* and *k_H_* values, and, hence, Equation (6) should be changed into the following form:

(23) $$S\left(t\right)=\int_{t_{0}}^{t_{1}}k_{T1}k_{H1}D_{0}{\left(\frac{t_{0}}{t}\right)}^{m}dt+\int_{t_{1}}^{t_{2}}k_{T2}k_{H2}D_{0}{\left(\frac{t_{0}}{t}\right)}^{m}dt+\cdots +\int_{t_{n}}^{t}k_{Tn}k_{Hn}D_{0}{\left(\frac{t_{0}}{t}\right)}^{m}dt$$

where *t_1_*, *t_2_*, … *t_n_* are different points in time. *k_Tn_* and *k_Hn_* are different coefficient under different temperature and humidity. Accordingly, the corrosion current density is also a piecewise function. According to the meteorological data of the national surface meteorological observation station, the probability distribution characteristics of the annual average temperature and humidity in Qingdao area for 50 years were calculated. The average annual temperature is normally distributed, the mean is 13.24 °C, and the standard deviation is 0.899 °C. The average annual humidity is normally distributed, the mean is 72.2%, and the standard deviation is 2.355%. The code for acceptance of constructional quality of concrete structures [59] stipulates the thickness error and geometric size error. The mean and standard deviation can be calculated assuming these parameters distribute normally. The statistical parameters of temperature and humidity together with other parameters which are treated as random variables are listed in Table 4.

The environment of coastal structures under chloride ingress in this paper are divided into the wave splashing zone, the coastal atmospheric zone, and the offshore atmospheric zone. The values of RH in the wave splashing zone and coastal atmospheric zone are relatively stable, hence, constant relative humidity was used instead of meteorological data in the calculation of chloride ingress. The values of relative humidity in the wave splashing zone and coastal atmospheric zone are 0.90 and 0.75, respectively.

### 3.3. Calculation Process

**Figure 4:**
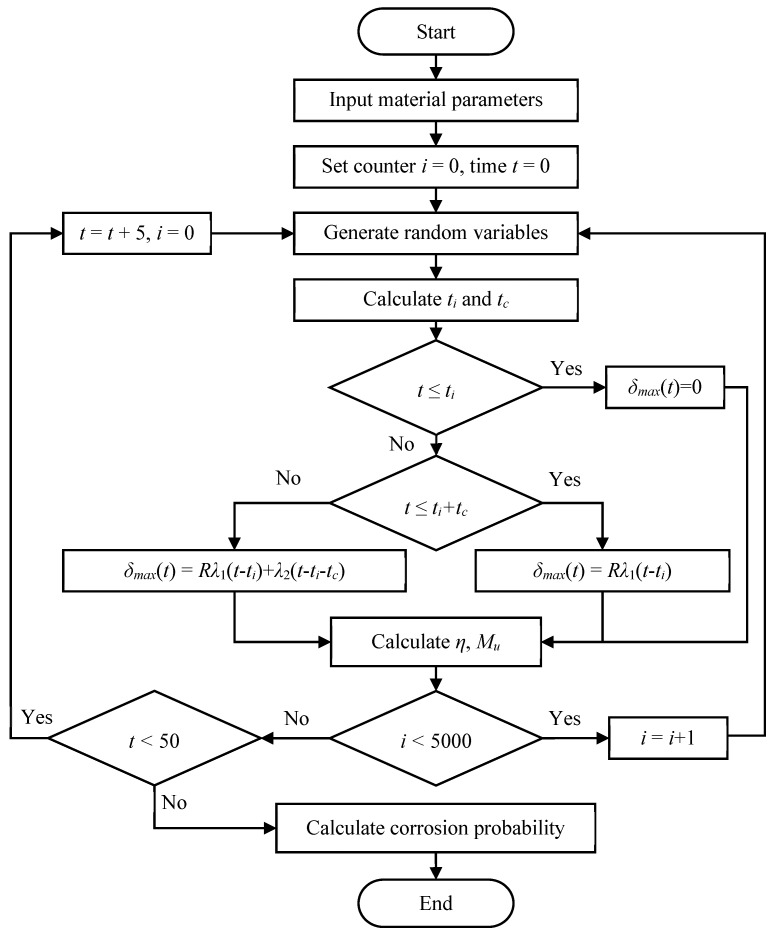
Flow chart of the program.

In this paper, the Monte Carlo method was applied to obtain the probability of corrosion. For the engineering structure, the designed service life is usually 50 years. In the calculation process, the whole service life was 100 years and was divided into one hundred equal parts and every period was one year. At each year, all the random variables were generated randomly according to the distribution which are shown in Table 4. Then the flexural strength, corrosion initiation time and other concerned values can be calculated. The above calculation was repeated *n* times at each time point. The corrosion frequency is the number of corrosion beams divided by *n*. According to Bernoulli’s law of large numbers, the probability can be obtained by the frequency at each time point. The probability converges to a value with the increase of *n* in the Monto Carlo method. In order to determine a reasonable number of repetitions, the initial corrosion probability of steel reinforcement within five years was calculated with 3000, 4000, and 5000 repetitions. Ten values of probability were calculated with each different repetition numbers. The variance of each group of data with different repetition numbers are shown in Table 5. As shown, the variance is pretty small when the repetition number is 5000. Thus, the calculation was repeated 5000 times at each time point. The calculation flow chart of chloride ingress is shown in Figure 4.

## 4. Results and Discussions

### 4.1. Probability of Corrosion

**Figure 5:**
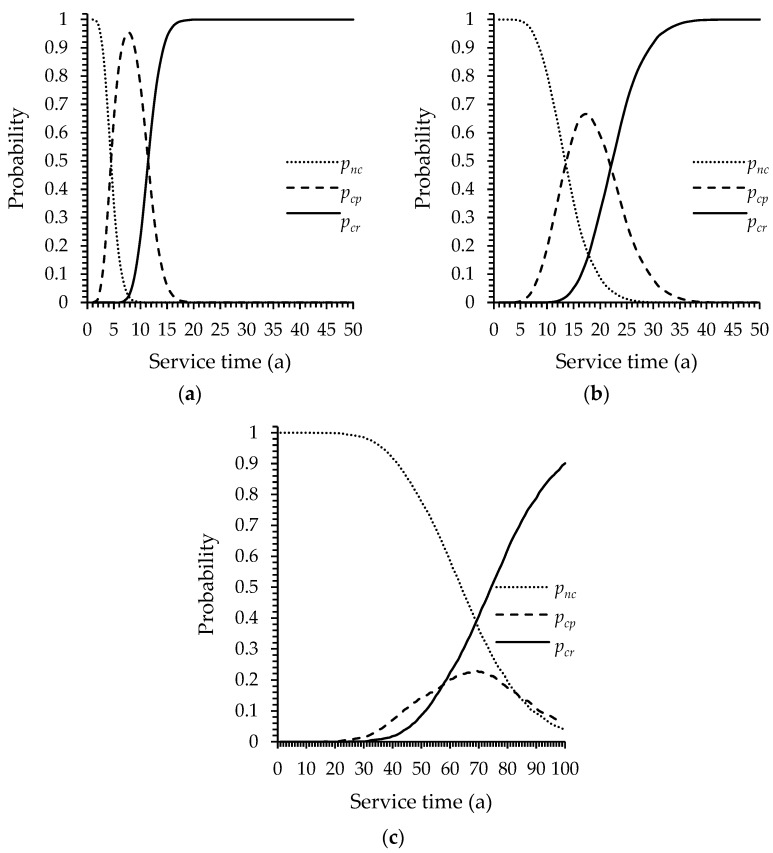
The corrosion probability of different area. (**a**) Wave splashing zone; (**b**) coastal atmospheric zone; (**c**) offshore atmospheric zone.

The probabilities calculated by the Monte Carlo method are shown in Figure 5. The reinforced beam in the calculation will stay in three conditions, which include non-corrosion, corrosion propagation, and concrete cover cracking. *p_nc_* is the probability of non-corrosion. *p_cp_* is the probability of corrosion propagation. *p_cr_* is the probability of concrete cover cracking. The sum of the three probability values is equal to 1. From Figure 5a, with the increase of service time, the probability of concrete cover cracking increases. The probability of steel bar corrosion in the first five years is about 0.5 in splash zone. The probability of non-corrosion reduces close to 0 in 10 years, which means most of corrosion initiation occur in 10 years for the beams in splash zone. After beams been in service for 20 years, the probability of concrete cover cracking reaches 1.

By comparing the corrosion probability in Figure 5a,b, it can be found that the probability of steel bar corrosion in coastal atmospheric zone is lower than that in splash zone in the same time. The year when *p_cr_* reaches 1 is postponed to the 35th year. The corrosion propagation period starts at five years and ends at about the 40th year, which means the variance of corrosion initiation has increased. This is due to the decrease of diffusion coefficient caused by the decrease of humidity, combined with the decrease of surface chloride concentration. A small change in the thickness of concrete cover can cause a drastic change in the corrosion initiation time, which makes the corrosion initiation time to be more sensitive to the variance of the concrete cover thickness.

Figure 5c shows the corrosion probability in the offshore atmospheric zone. The degree of corrosion is lower than that in other two regions. The probability of non-corrosion within 50 years is 0.77. After 40 years of service, the probability of the concrete cover cracking begins to increase rapidly. It can be observed that the degree of variation of corrosion increases obviously. The corrosion initiation time varies from 20 to 100 years and even beyond 100 years. One reason is the decrease of surface chloride concentration. In the offshore atmospheric zone, the surface chloride concentration is 3 kg/m^3^, which is lower than the other two zones. According to Equation (1), the concentration gradient is small and the driving force of diffusion is weak. Therefore, the corrosion initiation time is longer than other zones. The lower RH value is the other important reason. The chloride diffusion coefficient with a RH value of 75% is only half of that at saturation according to Equation (5) [26,44]. The corrosion initiation time, which is already longer than other zones, will be doubled. Hence, the dispersion of the initiation time is enhanced. This indicates that the effect of humidity on the corrosion probability is greater in areas with lower surface chloride concentration. However, it is very difficult to determine the value of RH in the offshore atmospheric zone, because the RH of environment in which the structure is located is greatly affected by rainfall and wind direction.

**Figure 6:**
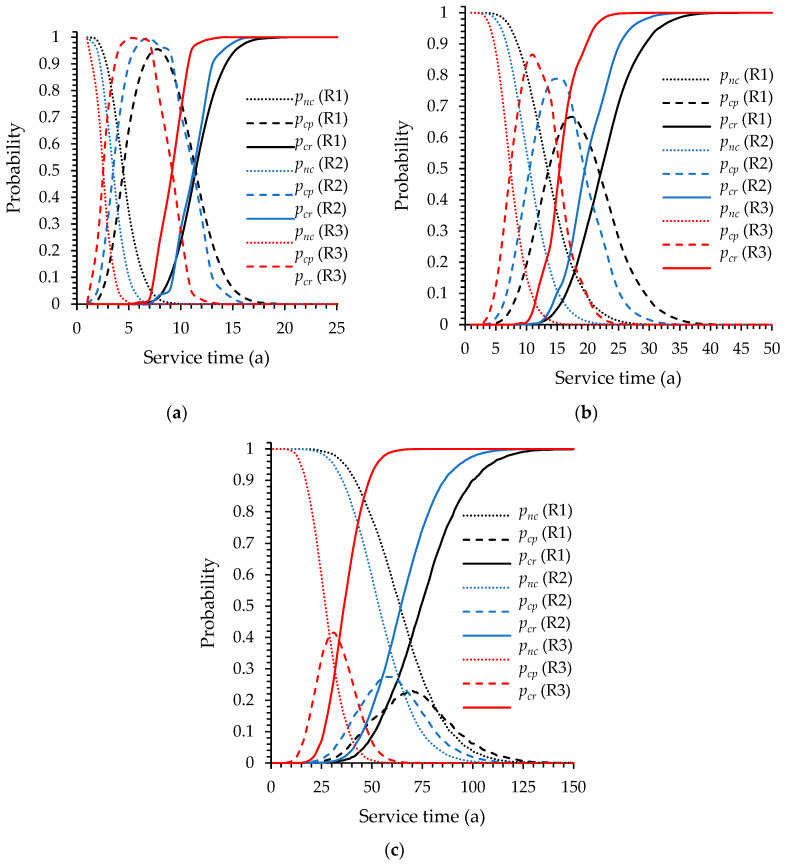
The corrosion probability of different area in different locations. (**a**) Wave splashing zone; (**b**) coastal atmospheric zone; (**c**) offshore atmospheric zone. R1 refers to region 1: Qingdao; R2 refers to region 2: Shanghai; R3 refers to region 3: Xiamen.

In order to compare the climatic conditions (temperature and humidity) of different locations, other two typical locations were calculated. Shanghai and Xiamen are two major ports in South China and temperature and humidity are higher than in North China. The average annual temperature and average annual relative humidity of Shanghai are 16.8 °C and 74.3%, respectively. The standard deviation of temperature and relative humidity are 1.103 °C and 2.13%, respectively. The average annual temperature and average annual relative humidity of Xiamen are 21.8 °C and 78.8%, respectively. The standard deviation of temperature and relative humidity are 0.912 °C and 2.54%, respectively. The results are shown in Figure 6. R1 refers to region 1: Qingdao; R2 refers to region 2: Shanghai; R3 refers to region 3: Xiamen. Higher temperature and RH value increase the corrosion probability. Especially, the cracking probability of the offshore atmospheric zone in Xiamen increase significantly. This corresponds to the discussion above, that is, temperature and humidity have a more obvious effect in the region with lower surface chloride concentration. This is similar to [60], which indicates that the corrosion initiation time reduced in the tropical environment.

### 4.2. Effect of Randomness of Temperature and Humidity on Corrosion Probability

The temperature fluctuates randomly in the different regions. When the temperature fluctuation is small, the average temperature can be treated as a constant. In order to understand the effect of temperature fluctuations on the probability of corrosion, the random variables of temperature with different assumptive standard deviations were introduced into the calculation. After the cracking of the concrete cover, the load bearing capacity will decrease rapidly in a short time. Therefore, the probability of concrete cover cracking is most concerned. For beams in the wave splashing zone, the randomness of humidity is not introduced into the calculation because of the RH is relatively stable in the wave splashing zone. The *p_cr_* of the wave splashing zone with different temperature mean values and standard deviation, and another group of *p_cr_*_,0_ which have the same temperature mean values but zero standard deviation, are calculated.

The *p_cr_* is a sequence of values arranged by time and each number represents the probability of cracking of each year in a range from 0 to 1. Hence, the *p_cr_* can be treated as a multidimensional vector and each dimension is the probability of cracking in a given year. The results of the calculation should be the same each time, but because the Monte Carlo method has a limited number of calculations, there will be small random fluctuations in the results. The Euclidean distance between the two vectors is used to measure the fluctuation as Equation (24). *x_i_* and *y_i_* represent the members of the two vectors, respectively:

(24) $$\mathrm{dist}=\;\sqrt{\sum {{(x}_{i}{-y}_{i})}^{2}}$$

The distance is distributed normally, which is verified by the Lilliefor test. The parameters of the normal distribution are estimated by the maximum likelihood method. The mean is 0.0384 and the standard deviation is 0.01465. This probability distribution of the vector distance between two same calculations is recorded as dist(0)~N(0.0384,0.0002). A total of 25 combinations of five different temperature means (8~25 °C) and five different standard deviations (0.5~2.5 °C) were calculated to analyze the effect of temperature randomness. The distance between vector *p_cr_* and vector *p_cr,_*_0_ is calculated for every combination and is recorded as dist(T, SD). Every combination is calculated 100 times, forming 100 values of distance, and the mean values are shown in Table 6. The probability distribution of distances calculated with different combinations may differ from the base distribution, dist(0). The mean values in Table 6 can only preliminarily determine whether the distance conforms to the distribution of dist(0). The Kolmogorov-Smirnov tests (K–S test) are conducted between each dist(T, SD) and dist(0) to evaluate the differences. The null hypothesis (H0) is that dist(T, SD) and dist(0) are from the same distribution. The alternative hypothesis (H1) is that dt(T, SD) and dist(0) are from different distributions. The K–S test results are also shown in Table 6. If the alternative hypothesis is accepted, it indicates that the randomness of temperature should be taken into account.

From Table 6, the effect of temperature fluctuation is related to the standard deviation of temperature. The distance between two vectors increase with the standard deviation, which means the difference between *p_cr_* and *p_cr,_*_0_ gets larger. When the standard deviation of temperature is greater than 1.5 °C, the alternative hypothesis is accepted. It indicates that the difference between *p_cr_* and *p_cr,_*_0_ is statistically significant. Therefore, the difference between *p_cr_* with temperature randomness and those without temperature randomness is insignificant when the standard deviation of the temperature is less than 1.5 °C in the wave splashing zone. In this condition, the temperature can be treated as a constant in the calculation of chloride ingress.

**Figure 7:**
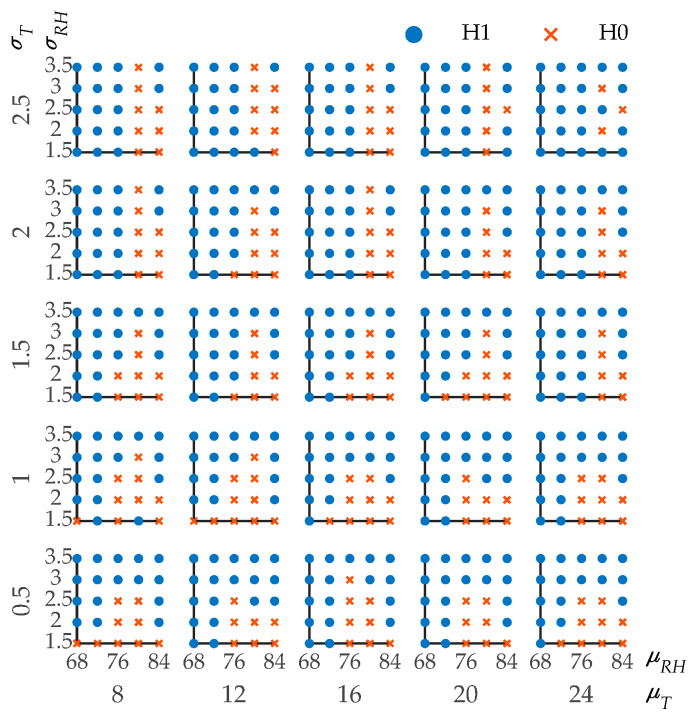
Hypothesis testing results of corrosion probability in the offshore atmospheric zone for all combinations.

For the structures in the offshore atmospheric zone, the ambient temperature and humidity fluctuate randomly at the same time. The effect of the humidity randomness should also be considered. The effect of the humidity randomness is considered using the same method as the temperature but the calculation becomes more complicated. Five different means of temperature, standard deviations of temperature, means of RH, and standard deviations of RH, which is a total of 625 combinations, are calculated. The means of temperature (*μ_T_*) are 8, 12, 16, 20, 24 °C. The standard deviations of temperature (*σ_T_*) are 0.5, 1, 1.5, 2, 2.5 °C. The means of RH (*μ_RH_*) are 68%, 72%, 76%, 80%, 84%. The standard deviations of RH (*σ_RH_*) are 1.5%, 2%, 2.5%, 3%, 3.5%. All of the 625 combinations are shown in Figure 7. A scatter plot matrix is applied to illustrate the test results of different combinations. Every single plot is a group of *μ_RH_* vs. *σ_RH_* with a certain combination of mean and standard deviation of temperature. The row and column represent *σ_T_* and *μ_T_*, respectively. H1 means that the calculation results of considering temperature and humidity randomness are statistically different from those without randomness.

In most combinations, the temperature and humidity randomness should be taken into account in the calculation process of *p_cr_*, especially when the *μ_RH_* is low. Obviously, the increase of *σ_RH_* will enhance the influence of randomness. From the results, the randomness should be considered in most cases where *σ_RH_* is greater than 3%. Most of the testing results of the combinations with *μ_RH_* less than 76% showed the rejection of the null hypothesis, which means that the randomness of the temperature and humidity have a significant impact. This is because the decrease of relative humidity will cause a significant decrease in the diffusion coefficient, which makes the chloride diffusion process more sensitive to random changes [61]. In [26,44,61], Equation (5) was used to calculate the effect of humidity on chloride diffusion. A small change in humidity will cause a considerable change in the diffusion coefficient of chloride ions based on Equation (5). There are limited studies focused on the randomness of humidity. In [62], a comprehensive model including humidity randomness was proposed and the results confirm that transfer mechanisms are very sensitive to environmental actions, especially the humidity. From the calculation results with 625 combinations of temperature and humidity, it is found that the influence of the humidity variation on corrosion probability is greater than the temperature. This is because the temperature has less influence on the chloride diffusion coefficient based on Equation (4) which was widely used in [27,28,60,62,63]. The same equation was used in [63] to study the effects of climate change on the corrosion probability of the infrastructure in Australia, the result showing that the temperature will increase the chloride diffusion coefficient and corrosion rate, but these increases will be relatively modest with relatively little influence on chloride-induced corrosion. From the calculation result in this paper, whether to consider the randomness is determined by both temperature and humidity.

In order to determine the threshold of parameters when the randomness should be considered, a binary logistic regression of 625 sets of data is conducted. The dependent variable, *y*, in the regression equation is 1 or 0. When *y* = 1, the alternative hypothesis is accepted and means that the randomness of temperature and humidity cannot be ignored. The prediction accuracy of H1 is 89.2%. The confusion matrix is shown in Table 7. The regression expression is shown as follows:

(25) $$p\left(y=1\right)={\left(1-e^{-0.038\mu_{T}-0.444\sigma_{T}+0.287\mu_{RH}-1.83\sigma_{RH}-17.352}\right)}^{-1}$$

where *μ_T_* and *σ_T_* are the mean and standard deviation of temperature (°C). *μ_RH_* and *σ_RH_* are the mean and standard deviation of the relative humidity (%). *P* is the probability of *y* = 1. The value of *p* can be obtained by substituting the temperature and humidity data of a certain location into Equation (25). When *p* > 0.5, the alternative hypothesis is accepted which means the randomness of temperature and humidity in this region should be considered.

### 4.3. Flexural Strength of Reinforced Concrete Beams

**Figure 8:**
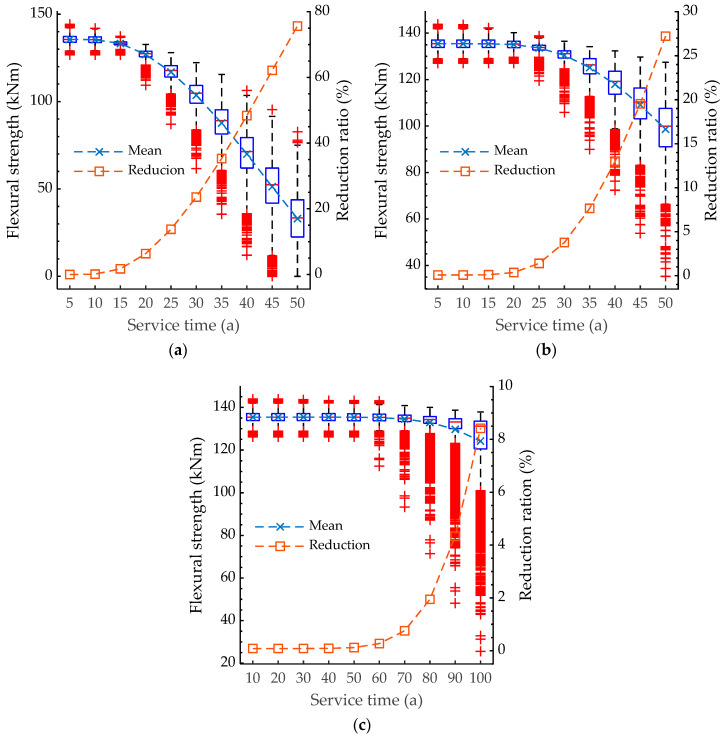
Flexural strength of reinforced concrete beam. (**a**) Wave splashing zone; (**b**) coastal atmospheric zone; (**c**) offshore atmospheric zone.

**Figure 9:**
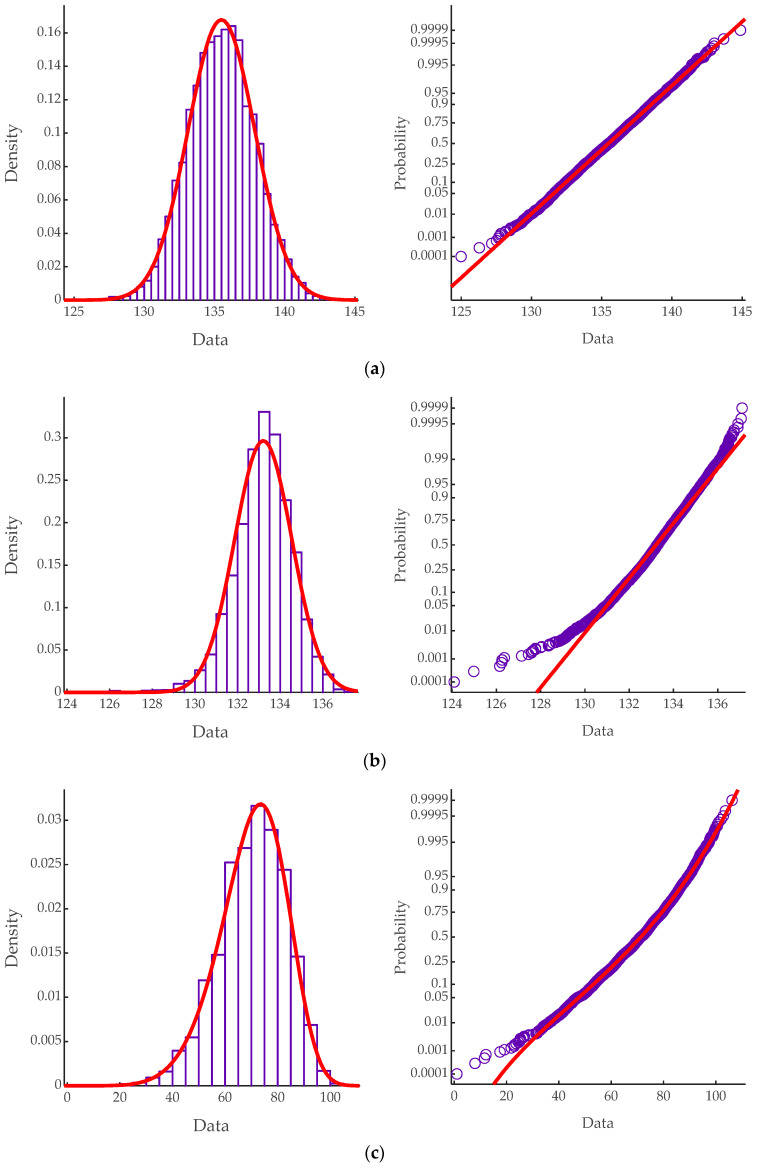
Probability density plots and probability plots of resistance distribution fitting for the wave splashing zone. (**a**) Distribution fitting of five-year flexural capacity against the normal distribution. (**b**) Distribution fitting of 15-year flexural capacity against the normal distribution. (**c**) Distribution fitting of 40-year flexural capacity against the Weibull distribution.

Figure 8 shows the boxplots of flexural strength of reinforced concrete beams varies with time. All the flexural strengths are plotted every five years for the wave splashing zone and coastal atmospheric zone. The reduction ratios of the mean flexural strength are plotted on the secondary axis. For the offshore atmospheric zone, the flexural strengths are plotted every 10 years. The flexural strength of the beam designed in Table 2 is 135.09 kN·m without considering the randomness of the geometry dimensions and concrete strength. All the mean values in Figure 8a,b are close to the median and decrease over time. When the service time reaches 50 years, the mean flexural capacity decreases by 75.5% and 26.9% for the wave splashing zone and coastal atmospheric zone, respectively. The mean flexural strength decreases barely over the first 70 years of service for the offshore atmospheric zone, but more outliers appear. In the early years of service, the flexural strength distribution is symmetrical. With the development of corrosion, the flexural strength distribution is no longer symmetrical, and more and more low flexural strength outliers appear. The interquartile range increases over service time. By comparing Figure 6 and Figure 9, it is observed that when most beams are in the corrosion propagation period, the flexural strength distribution remains symmetrical but the number of outliers in the lower side increases. When beams are in the concrete cover cracking period, the flexural strength begins to decrease rapidly and distribution begins to shift to the lower side.

In order to determine that the flexural strength conforms to which probability distribution, the normal distribution, extreme value distribution, and Weibull distribution are used for parameter estimation, respectively. Kolmogorov–Smirnov tests are carried out to verify the goodness of fit. Test results are shown in Table 8. It can be observed that the change trend of flexural strength is similar and the difference is the time point when resistance begin to decrease. Some of the flexural strength of the splash area decreases to 0 in 50 years, covering all stages of flexural strength change. Hence, the test results of wave splashing zone are taken as an example. The significance level of the K–S test is 0.05. K–S test results show that the flexural strength conforms to normal distribution in the first five years of service. In 20–40 years, the flexural strength distribution is asymmetric and conforms to the extreme value distribution or Weibull distribution. Additionally, the *p*-value indicates that the distribution fitting against the Weibull distribution has a better goodness of fit.

There are three stages in the deterioration process of reinforced concrete structures due to steel corrosion. The first stage is dominated by the penetration of chloride, during which chloride ions diffuse through concrete toward the reinforcing bars [13]. In this stage, the chloride concentrations on the steel bar surface do not reach the threshold and the corrosion will not occur [64]. From the flow chart in Figure 4, this stage is determined by the first decision block (*t* < *t_i_*). In this stage, the corrosion depth is zero due to the algorithm, therefore, the flexural strength remains the same. The distribution conforms to the normal distribution, which is determined by the randomness of the geometric dimension parameters and concrete strength. This period lasts for five years and corresponds to Figure 5a. As shown in Figure 8, the probability of low flexural strength increases and the distribution becomes asymmetric with the increase of time. During this period, the flexural strength distribution is neither a normal nor Weibull distribution according to the K–S test results, which is from 10 to 15 years. The corrosion current density increases rapidly after the concrete cover cracks [15,38]. As a result, the flexural strength begins to decline rapidly according to Equations (15)–(22). According to the K–S test results, the distribution converts from a normal distribution to a Weibull distribution. This period lasts from 20 to 40 years and corresponds to the concrete cracking period in Figure 5a. In the last ten years in service time, the flexural strength distribution becomes symmetrical again due to the serious corrosion of most beams, which is similar to the result in [65].

Due to the influence of corrosion, the shape of the flexural strength distribution will change, which can be roughly divided into two stages. It conforms to the normal distribution in the early stage of service and the Weibull distribution after concrete cracking. Figure 9 shows a group of probability density plots at three typical time points to illustrate the goodness of fit. As shown in Figure 9a,c, the distributions are fitting well at five years and 40 years and the null hypothesis is accepted in the K–S tests. Although the null hypothesis is rejected in the K–S test at 15 years, the flexural strength approximately obeys the normal distribution as shown in Figure 8b. In the calculation of structural reliability, in order to simplify the calculation procedure, the flexural strength can be regarded as following a normal distribution before concrete cracking and following a Weibull distribution after concrete cracking.

### 4.4. Effect of Randomness of Temperature and Humidity on Flexural Strength

The influence of temperature randomness on the flexural strength of beam in the wave splashing zone is evaluated. Two groups of flexural strength of 50 years are calculated with and without temperature randomness. The calculation is repeated 5000 times at each time point. Each sample contains 5000 data points. The influence of temperature randomness was verified by the K–S test to determine whether the two samples are from the same population. When the alternative hypothesis is accepted, it indicates that the two samples come from different populations, that is, temperature randomness will cause the change of flexural strength distribution and, hence, the randomness cannot be ignored. Table 9 shows which years have statistically significant differences under different combinations of means and variances. When the temperature standard deviation is less than 1 °C, the temperature randomness has no significant effect on flexural strength. The period during which the temperature randomness makes a difference is usually in the middle of service, such as 15~40 years. In the early stage, chloride ions diffuse into concrete and do not accumulate to a critical value on the surface of steel bars [13]. This stage is called the initiation stage of corrosion [26,41,64]. In the Life-365 program [41] and this paper, the corrosion will not occur during this period and, hence, the flexural strength remain the same. Therefore, the K–S tests give the results that the flexural strengths are from the same distribution. From Equations (1)–(10) and the algorithm flow chart in Figure 4, the temperature randomness will not affect the flexural strength but will affect the length of the corrosion initiation stage. This is similar to the results in studies [15,66]. From the calculation results shown in Figure 8a, a large number of flexural strengths decrease to 0 in 50 years. This situation is not likely to happen in real structures because the beam will collapse at a certain corrosion loss before its flexural strength drops to zero. In [67], it was found that the maximum corrosion loss in a reinforcing bar conditional on beam collapse was no more than 16%, which corresponds to being in service for about 25 years in this paper. Mathematically, many zero flexural strengths appear, which means many of the same elements in the K–S test leads to the result that the flexural strengths are from the same distribution given by the K–S test.

**Figure 10:**
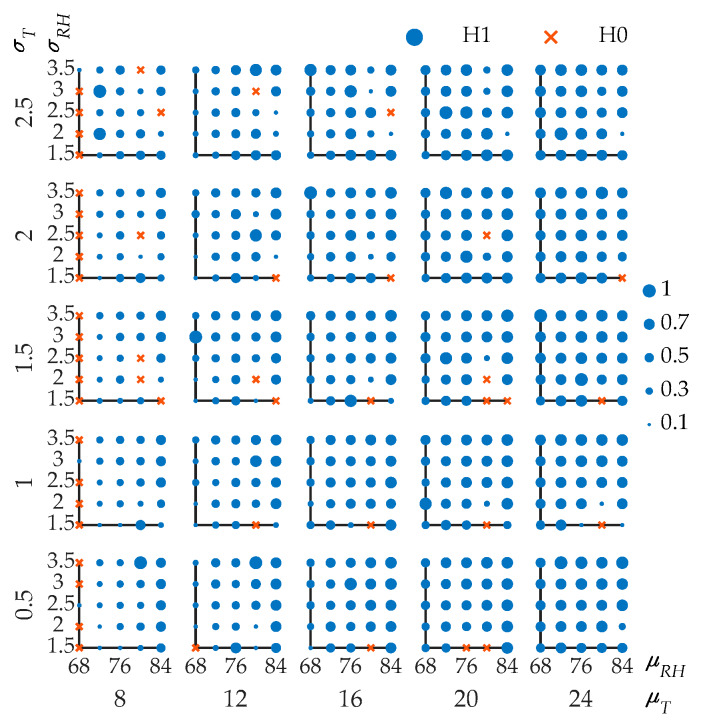
Hypothesis testing results of flexural strength in the offshore atmospheric zone for all combinations.

Temperature and humidity change randomly at the same time in the offshore atmospheric zone. Five different means of temperature, standard deviations of temperature, means of RH, and standard deviations of RH comprise a total of 625 combinations to be calculated. The means of temperature (*μ_T_*) are 8, 12, 16, 20, 24 °C. The standard deviations of temperature (*σ_T_*) are 0.5, 1, 1.5, 2, 2.5 °C. The means of RH (*μ_RH_*) are 68%, 72%, 76%, 80%, 84%. The standard deviations of RH (*σ_RH_*) are 1.5%, 2%, 2.5%, 3%, 3.5%. Two groups of flexural strengths are calculated with and without temperature and humidity randomness, respectively. Each group contains 100 years and every time point has 5000 flexural strengths. The K–S test is conducted for two groups of flexural strength to verify whether they are from the same population. This process is applied to every combination. It is difficult to give all the information of the results. Similar to Figure 7, all combinations are given in the form of scatter plots in Figure 10, the area of each point represents a ratio of the number of years affected by randomness to the total number of years. The randomness of the temperature and humidity in most combinations will cause the distribution of flexural strength to change. Therefore, it suggests that the temperature and humidity randomness should be taken into account in the calculation of chloride ingress in the offshore atmospheric zone. However, the RH value in this region is affected by wind and rain and the accumulating process of surface chloride concentration in this region is more complicated. The accurate flexural strength distribution of the offshore atmospheric zone requires further experimental studies.

## 5. Conclusions

The influence of temperature and humidity on chloride ion diffusion was analyzed. The influence of the randomness of temperature and humidity was introduced by treating the annual average temperature and humidity as piecewise functions. Taking temperature, humidity, geometric size, and concrete strength as random variables, the corrosion probability and the distribution of flexural strength of a typical design beam was obtained by the Monte Carlo method. Results show that the corrosion probabilities of the wave splashing zone, coastal atmospheric zone, and offshore atmospheric zone are very different. This is mainly due to the difference in the concentration of chloride ions on the surface. The RH value significantly affects the diffusion coefficient and the corrosion probability of the offshore atmospheric zone is less than that in other two zones at the same time. The randomness of temperature and humidity will affect the cracking probability. The difference between *p_cr_* with temperature randomness and those without temperature randomness is insignificant when the standard deviation of the annual average temperature is less than 1.5 °C in the wave splashing zone. In this condition, the temperature can be treated as a constant throughout the service life in the calculation of chloride ingress. For the beam in the offshore atmospheric zone, the influence of humidity on the cracking probability is greater than that of the temperature. A binary logistic regression formula is given to predict whether the randomness of temperature and humidity should be considered.

The flexural strength decreases rapidly after concrete cover cracking. The flexural strength distribution conforms to the normal distribution in the early stage of service and Weibull distribution after concrete cracking. When the annual average temperature standard deviation is less than 1 °C, the temperature randomness has no significant effect on the flexural strength of beams in the wave splashing zone. The period during which the temperature randomness makes a difference is usually in the middle of service. For the beam in the offshore atmospheric zone, the temperature and humidity randomness should be taken into account in the calculation of chloride ingress. This method can be used to predict the corrosion probability and the flexural strength of the beam or other reinforced concrete member of a given design size.

## Figures and Tables

**Table 1:** Comparison between experimental values and estimated values.

W/B	FA (%)	Chloride Diffusion Time (d)	Temperature (℃)	RH (%)	Age (d)	Experimental Values	Estimated Values	Estimated/ Experimental
0.6	0	180	22	0.95	28	3.73 ^1^	3.75	1.01
0.6	0	180	22	0.86	28	2.02 ^1^	2.4	1.19
0.6	0	180	22	0.75	28	0.67 ^1^	0.63	0.94
0.6	0	28	22	1	28	16.45 ^2^	18.47	1.12
0.55	0	28	22	1	28	15.08 ^2^	14.02	0.93
0.45	30	28	22	1	28	12.4 ^2^	11.07	0.89
0.4	20	28	20	1	28	8.5 ^3^	7.94	0.93
0.4	30	28	20	1	28	9.4 ^3^	8.04	0.84
0.48	0	56	5	1	56	5.98 ^4^	4.87	0.81
0.6	0	56	40	1	56	29.51 ^4^	29.6	1.003
0.35	0	56	5	1	56	5.65 ^4^	5.24	0.93

^1^ Data source [45]. ^2^ Data source [46]. ^3^ Data source [48]. ^4^ Data source [47].

**Table 2:** Material parameters of reinforced concrete beam in calculation.

Pameter	Value
Water cement ratio	0.4
Compressive strength of concrete cube	40 MPa
Cement dosage	400 kg/m^3^
Concrete cover thickness	50 mm
Diameter of steel bar	16 mm
Total section area of bottom steel bar	804 mm^2^
Beam section height	500 mm
Beam section width	250 mm

**Table 3:** Recommendations for the value of *C_s_* (kg/m^3^) in different zone.

Source	Wave Splashing Zone	Coastal Atmospheric Zone	Offshore Atmospheric Zone
ACI-365	18.4	23(2.3) ^1^	13.8(0.92) ^1^
JSCE	14.95	10.35	2.3
DuraCrete	12.42	4.11	4.11
GB/T51355-2019	17	11.5	2.57
Bamforth	18	12	6
McGee	-	2.95	1.69

^1^ The value in brackets is the annual cumulative amount.

**Table 4:** Statistical parameters and probability distribution types of variables.

Random Variable	Mean	Standard Deviation	Distribution
Average annual temperature (°C)	13.24	0.899	Normal
Average annual relative humidity (%)	72.2	2.355	Normal
Compressive strength of concrete (MPa)	44.11	2.5	Normal
Concrete cover thickness (mm)	51.5	6.64	Normal
Section height (mm)	500	3.03	Normal
Section breadth (mm)	250	3.03	Normal

**Table 5:** Variance of initial corrosion probability of reinforcement with different repetition numbers.

Area	Repetition number		
	3000	4000	5000
Wave splashing zone	3.36 × 10^−5^	2.33 × 10^−5^	1.4 × 10^−5^
Coastal atmospheric zone	1.61 × 10^−4^	3.99 × 10^−5^	1.46 × 10^−5^
Offshore atmospheric zone	2.92 × 10^−5^	2.49 × 10^−5^	1.64 × 10^−5^

**Table 6:** The mean distances between vector *p_cr_* and *p_cr,_*_0_ with different means and SDs.

Standard Deviation (°C)	Mean Temperature (°C)				
	8	12	16	20	24
0.5	0.0372(H0) ^1^	0.0320(H0)	0.0269(H0)	0.0239(H0)	0.0229(H0)
1	0.0375(H0)	0.0325(H0)	0.0317(H0)	0.0274(H0)	0.0235(H0)
1.5	0.0429(H1) ^2^	0.0365(H1)	0.0342(H1)	0.0279(H1)	0.0256(H0)
2	0.0535(H1)	0.0551(H1)	0.0442(H1)	0.0381(H1)	0.0327(H1)
2.5	0.0822(H1)	0.0714(H1)	0.0597(H1)	0.0515(H1)	0.0443(H1)

^1,2^ The H0 or H1 in brackets represents the accepted hypothesis.

**Table 7:** The confusion matrix of the prediction model.

		Predictive value		Precision
		1	0	
True Value	1	388	47	89.2%
	0	69	121	-
Recall		84.9%	-	81.4% ^1^

^1^ Accuracy.

**Table 8:** The *p*-values of the K–S tests against different types of distribution for the wave splashing zone.

Service Time	Normal Distribution	Extreme Value Distribution	Weibull Distribution	Mean ^1^	Standard Deviation ^1^	A ^2^	B ^2^
5	0.862	1.27 × 10^−17^	2.08 × 10^−15^	135.49	2.37	-	-
10	0.045	4.06 × 10^−10^	6.91 × 10^−9^	135.32	2.23	-	-
15	2.60 × 10^−6^	5.23 × 10^−15^	3.92 × 10^−14^	133.20	1.34	-	-
20	9.25 × 10^−24^	0.677	0.830	-	-	128.10	63.96
25	3.71 × 10^−17^	0.217	0.699	-	-	119.21	29.19
30	9.19 × 10^−13^	0.012	0.335	-	-	107.17	16.32
35	2.98 × 10^−9^	3.03 × 10^−4^	0.290	-	-	92.40	10.09
40	9.87 × 10^−8^	2.41 × 10^−6^	0.570	-	-	75.43	6.44
45	3.06 × 10^−5^	1.71 × 10^−8^	0.011	51.63	14.50	-	-
50	0.034	3.39 × 10^−14^	4.13 × 10^−9^	33.32	14.83	-	-

^1^ Parameter estimation using normal distribution fitting. ^2^ Parameter estimation using Weibull distribution fitting.

**Table 9:** The years in which the temperature randomness makes a significant difference.

Standard Deviation (°C)	Mean Temperature (°C)				
	8	12	16	20	24
0.5	-	-	-	-	-
1	-	-	-	-	-
1.5	-	20	15–20	-	10–15
2	20–50	20–30	10–20	15–45	10–40
2.5	15–50	15–50	15–50	10–45	5–40

## References

[1] Li L.-Y., Xia J., Lin S.-S. (2012). A multi-phase model for predicting the effective diffusion coefficient of chlorides in concrete. Constr. Build. Mater..

[2] Abyaneh S.D., Wong H.S., Buenfeld N.R. (2013). Modelling the diffusivity of mortar and concrete using a three-dimensional mesostructure with several aggregate shapes. Comput. Mater. Sci..

[3] Zhang W.-M., Ba H.-J. (2013). Effect of silica fume addition and repeated loading on chloride diffusion coefficient of concrete. Mater. Struct..

[4] Liu J., Tang K., Pan D., Lei Z., Wang W., Xing F. (2014). Surface chloride concentration of concrete under shallow immersion conditions. Materials.

[5] Zhang J., Zhou X.-Z., Zheng J.-J., Ye H.-L., Yang J. (2020). Experimental investigation and analytical modeling of chloride diffusivity of fly ash concrete. Materials.

[6] Böhni H. (2005). Corrosion in Reinforced Concrete Structures.

[7] Masi A., Digrisolo A., Santarsiero G. (2019). Analysis of a large database of concrete core tests with emphasis on within-structure variability. Materials.

[8] Masi A., Digrisolo A., Santarsiero G., Matsuda K., Pa P.S., Yun W. (2014). Concrete strength variability in italian rc buildings: analysis of a large database of core tests. Advance Materials Development and Applied Mechanics.

[9] Kilinc K., Celik A.O., Tuncan M., Tuncan A., Arslan G., Arioz O. (2012). Statistical distributions of in situ microcore concrete strength. Constr. Build. Mater..

[10] Celik A.O., Kilinc K., Tuncan M., Tuncan A. (2012). Distributions of compressive strength obtained from various diameter cores. Aci Mater. J..

[11] Xiang T., Zhao R. (2007). Reliability evaluation of chloride diffusion in fatigue damaged concrete. Eng. Struct..

[12] Sarveswaran V., Roberts M.B. (1999). Reliability analysis of deteriorating structures—The experience and needs of practising engineers. Struct. Saf..

[13] Kong J.S., Ababneh A.N., Frangopol D.M., Xi Y. (2002). Reliability analysis of chloride penetration in saturated concrete. Probabilistic Eng. Mech..

[14] Jung W.-Y., Yoon Y.-S., Sohn Y.-M. (2003). Predicting the remaining service life of land concrete by steel corrosion. Cem. Concr. Res..

[15] Vu K.A.T., Stewart M.G. (2000). Structural reliability of concrete bridges including improved chloride-induced corrosion models. Struct. Saf..

[16] Liang M.T., Wang K.L., Liang C.H. (1999). Service life prediction of reinforced concrete structures. Cem. Concr. Res..

[17] Oh B.H., Jang S.Y. (2004). Prediction of diffusivity of concrete based on simple analytic equations. Cem. Concr. Res..

[18] Kassir M.K., Ghosn M. (2002). Chloride-induced corrosion of reinforced concrete bridge decks. Cem. Concr. Res..

[19] Funahashi M. (1990). Predicting corrosion-free service life of a concrete structure. ACI Mater. J..

[20] Hooton R.D., Geiker M.R., Bentz E.C. (2002). Effects of curing on chloride ingress and implications on service life. ACI Mater. J..

[21] West R.E., Hime W.G. (1985). Chloride profiles in salty concrete. Mater. Perform..

[22] Collepardi M., Marcialis A., Turriziani R. (1972). Penetration of chloride ions into cement pastes and concretes. J. Am. Ceram. Soc..

[23] Tang L., Nilsson L.-O. (1992). Chloride diffusivity in high strength concrete at different ages. Nord. Concr. Res..

[24] Thomas M.D., Bamforth P.B. (1999). Modelling chloride diffusion in concrete: Effect of fly ash and slag. Cem. Concr. Res..

[25] Liu Q.F., Hu Z., Lu X.Y., Yang J., Azim I., Sun W.Z. (2020). Prediction of chloride distribution for offshore concrete based on statistical analysis. Materials.

[26] Saetta A.V., Scotta R.V., Vitaliani R.V. (1993). Analysis of chloride diffusion into partially saturated concrete. ACI Mater. J..

[27] Page C., Short N., El Tarras A. (1981). Diffusion of chloride ions in hardened cement pastes. Cem. Concr. Res..

[28] Xi Y., Bažant Z.P. (1999). Modeling chloride penetration in saturated concrete. J. Mater. Civ. Eng..

[29] Liu Q.F., Yang J., Xia J., Easterbrook D., Li L.Y., Lu X.Y. (2015). A numerical study on chloride migration in cracked concrete using multi-component ionic transport models. Comput. Mater. Sci..

[30] Liu Q.F., Easterbrook D., Yang J., Li L.Y. (2015). A three-phase, multi-component ionic transport model for simulation of chloride penetration in concrete. Eng. Struct..

[31] Liu Q.F., Feng G.L., Xia J., Yang J., Li L.Y. (2018). Ionic transport features in concrete composites containing various shaped aggregates: A numerical study. Compos. Struct..

[32] Liu Q.F., Easterbrook D., Li L.Y., Li D.W. (2017). Prediction of chloride diffusion coefficients using multi-phase models. Mag. Concr. Res..

[33] Jiang W.Q., Shen X.H., Hong S.X., Wu Z.Y., Liu Q.F. (2019). Binding capacity and diffusivity of concrete subjected to freeze-thaw and chloride attack: A numerical study. Ocean Eng..

[34] Jiang W.Q., Shen X.H., Xia J., Mao L.X., Yang J., Liu Q.F. (2018). A numerical study on chloride diffusion in freeze-thaw affected concrete. Constr. Build. Mater..

[35] Shen X.H., Jiang W.Q., Hou D.S., Hu Z., Yang J., Liu Q.F. (2019). Numerical study of carbonation and its effect on chloride binding in concrete. Cem. Concr. Compos..

[36] Shen X.H., Liu Q.F., Hu Z., Jiang W.Q., Lin X.S., Hou D.S., Hao P. (2019). Combine ingress of chloride and carbonation in marine-exposed concrete under unsaturated environment: A numerical study. Ocean Eng..

[37] Bazant M., Zdenek P. (1979). Physical model for steel corrosion in concrete sea structures—Application. J. Struct. Div..

[38] Liu T., Weyers R. (1998). Modeling the dynamic corrosion process in chloride contaminated concrete structures. Cem. Concr. Res..

[39] Zhang L., Niu D., Wen B., Luo D. (2019). Concrete protective layer cracking caused by non-uniform corrosion of reinforcements. Materials.

[40] Val D.V., Melchers R.E. (1997). Reliability of deteriorating RC slab bridges. J. Struct. Eng..

[41] Thomas M.D., Bentz E.C. (2002). Computer program for predicting the service life and life-cycle costs of reinforced concrete exposed to chlorides. Life365 Manual, SFA.

[42] Guzmán S., Gálvez J.C., Sancho J.M. (2011). Cover cracking of reinforced concrete due to rebar corrosion induced by chloride penetration. Cem. Concr. Res..

[43] Oh B.H., Jang S.Y. (2007). Effects of material and environmental parameters on chloride penetration profiles in concrete structures. Cem. Concr. Res..

[44] Na O., Cai X.-C., Xi Y. (2017). Corrosion prediction with parallel finite element modeling for coupled hygro-chemo transport into concrete under chloride-rich environment. Materials.

[45] de Vera G., Climent M.A., Viqueira E., Antón C., Andrade C. (2007). A test method for measuring chloride diffusion coefficients through partially saturated concrete. Part II: The instantaneous plane source diffusion case with chloride binding consideration. Cem. Concr. Res..

[46] Spiesz P., Brouwers H. (2013). The apparent and effective chloride migration coefficients obtained in migration tests. Cem. Concr. Res..

[47] Yuan Q., Shi C., De Schutter G., Audenaert K. (2009). Effect of temperature on transport of chloride ions in concrete. Concrete Repair, Rehabilitation and Retrofitting II.

[48] Alexandre Bogas J., Gomes A. (2015). Non-steady-state accelerated chloride penetration resistance of structural lightweight aggregate concrete. Cem. Concr. Compos..

[49] MOHURD (2019). Standard for Durability Assessment of Existing Concrete Structures.

[50] Gonzalez J., Andrade C., Alonso C., Feliu S. (1995). Comparison of rates of general corrosion and maximum pitting penetration on concrete embedded steel reinforcement. Cem. Concr. Res..

[51] CECS (2007). Standard for Durability Assessment of Concrete Structures.

[52] MOHURD (2011). Code for Design of Concrete Structures.

[53] Sagues A.A., Kranc S., Presuel-Moreno F., Rey D., Torres-Acosta A., Yao L. (2001). Corrosion Forecasting for 75-Year Durability Design of Reinforced Concrete.

[54] Fluge F. Environmental loads on coastal bridges. Proceedings of the International Conference on Repair of Concrete Structures. From Theory to Practice in a Marine Environment.

[55] Holand I. (1998). Concrete exposure stations in Norway-Anoverview. NCR Publ..

[56] Engelund S., Edvardsen C., Mohr L. (2000). General Guidelines for Durability Design and Redesign: DuraCrete—Probabilistic Performance Based Durability Design of Concrete Structures.

[57] Bamforth P. (1999). The derivation of input data for modelling chloride ingress from eight-year UK coastal exposure trials. Mag. Concr. Res..

[58] McGee R. (1999). Modelling of durability performance of Tasmanian bridges. ICASP8 Appl. Stat. Probab. Civ. Eng..

[59] MOHURD (2015). Code for Acceptance of Constructional Quality of Concrete Structures.

[60] Bastidas-Arteaga E., Chateauneuf A., Sánchez-Silva M., Bressolette P., Schoefs F. (2010). Influence of weather and global warming in chloride ingress into concrete: A stochastic approach. Struct. Saf..

[61] Bažant Z.P., Najjar L.J. (1971). Drying of concrete as a nonlinear diffusion problem. Cem. Concr. Res..

[62] Bastidas-Arteaga E., Chateauneuf A., Sánchez-Silva M., Bressolette P., Schoefs F. (2011). A comprehensive probabilistic model of chloride ingress in unsaturated concrete. Eng. Struct..

[63] Stewart M.G., Wang X., Nguyen M.N. (2011). Climate change impact and risks of concrete infrastructure deterioration. Eng. Struct..

[64] Val D.V., Stewart M.G. (2009). Reliability assessment of ageing reinforced concrete structures—Current situation and future challenges. Struct. Eng. Int..

[65] Ahsana P.V., Rao K.B., Anoop M.B. (2015). Stochastic analysis of flexural strength of RC beams subjected to chloride induced corrosion. Mater. Res. Ibero Am. J. Mater..

[66] Bhargava K., Mori Y., Ghosh A.K. (2011). Time-dependent reliability of corrosion-affected RC beams. Part 2: Estimation of time-dependent failure probability. Nucl. Eng. Des..

[67] Stewart M.G., Al-Harthy A. (2008). Pitting corrosion and structural reliability of corroding RC structures: Experimental data and probabilistic analysis. Reliab. Eng. Syst. Saf..

